# Improved secretory expression and characterization of thermostable xylanase and *β*-xylosidase from *Pseudothermotoga thermarum* and their application in synergistic degradation of lignocellulose

**DOI:** 10.3389/fbioe.2023.1270805

**Published:** 2023-09-18

**Authors:** Jinkang Chen, Hao Qin, Chaoqun You, Lingfeng Long

**Affiliations:** ^1^ Key Laboratory of Industrial Biotechnology, Ministry of Education, School of Biotechnology, Jiangnan University, Wuxi, China; ^2^ Eco-Materials and Renewable Energy Research Center (ERERC), College of Engineering and Applied Sciences, Nanjing University, Nanjing, Jiangsu, China; ^3^ Little Swan Electric Co., Ltd., Midea Group, Wuxi, China; ^4^ Jiangsu Key Lab for the Chemistry and Utilization of Agro-Forest Biomas, College of Chemical Engineering, Nanjing Forestry University, Nanjing, China

**Keywords:** xylanase, xylosidase, secretory expression, molecular docking, saccharification

## Abstract

Xylanase and *β*-xylosidase are the key enzymes for hemicellulose hydrolysis. To further improve hydrolysis efficacy, high temperature hydrolysis with thermostable hemicellulases showed promise. In this study, thermostable xylanase (Xyn) and *β*-xylosidase (XynB) genes from *Pseudothermotoga thermarum* were cloned and secretory expressed in *Bacillu subtilis*. Compared with *Escherichia coli* expression host, *B. subtilis* resulted in a 1.5 time increase of enzymatic activity for both recombinant enzymes. The optimal temperature and pH were 95°C and 6.5 for Xyn, and 95°C and 6.0 for XynB. Thermostability of both recombinant enzymes was observed between the temperature range of 75–85°C. Molecular docking analysis through AutoDock showed the involvement of Glu525, Asn526, Trp774 and Arg784 in Xyn-ligand interaction, and Val237, Lys238, Val761 and Asn76 in XynB-ligand interaction, respectively. The recombinant Xyn and XynB exhibited synergistic hydrolysis of beechwood xylan and pretreated lignocellulose, where Xyn and XynB pre-hydrolysis achieved a better improvement of pretreated lignocellulose hydrolysis by commercial cellulase. The observed stability of the enzymes at high temperature and the synergistic effect on lignocellulosic substrates suggested possible application of these enzymes in the field of saccharification process.

## 1 Introduction

A typical lignocellulosic biorefinery involves an essential enzymatic hydrolysis step, converting the lignocellulosic biomass into sugar intermediates by using enzyme cocktail (Long et al., 2022). To compromise the properties of most commercial enzymes, the current enzymatic hydrolysis frequently carries out at around 50°C. However, hydrolysis at mild temperature either requires a large amount of enzymes or takes a long time, challenging the economy of the bioconversion process (van der Zwan et al., 2017). To further improve the hydrolysis efficacy, enzymatic hydrolysis at higher temperature (80–90°C) is a viable strategy to speed up the catalytic reaction, enhance mass transfer, and decrease the slurry viscosity. Additionally, high temperature hydrolysis could sterilize the hydrolysate, making for the downstream fermentation process (Viikari et al., 2007; Chatterjee et al., 2015; Long et al., 2018). Therefore, high temperature enzymatic hydrolysis and the accompanying thermostable enzymes have been gained increasing attention in lignocellulose biorefinery.

Pretreatment is firstly required to break down the recalcitrant structure of lignocellulose and fractionate its main components (cellulose, hemicellulose and lignin) (Tian et al., 2022). In order to increase the cellulose accessibility and to avoid sugar lose during pretreatment process, mild-severity pretreatment is often conducted. Under these milder pretreatment conditions, some hemicellulose (mainly xylan in hardwood and annual plants) remains in insoluble fractions, limiting the extent and the rate of cellulose hydrolysis (Zhai et al., 2016). Recent works have shown that xylan degradation could significantly increase cellulose accessibility and fiber swelling, and thus greatly improve the hydrolytic efficiency. For example, the results by Jin et al. (2019) showed that xylan content was the key accelerant for cellulose conversion. After removing ∼20% xylan from the substrate, a ∼30% increase in hydrolysis efficiency (Jin et al., 2019). In addition, a recent study by Wu et al. (2020) showed that the removal of around 30% of the xylan from alkali pretreated corn stove could resulted in a large increase in cellulose accessibility and substrate swelling (65% increase in water retention value and 43% increase in Direct Orange dye adsorption) when compared to the control sample (Wu et al., 2020).

Unlike cellulose, which has a clearly defined linear structure, xylan is a heterogeneous branched polysaccharide with a *β*-1,4-xylosyl backbone and different side-chain residues, such as L-arabinofuranosyl, D-glucuronic and O-acetyl (Long et al., 2020b). Given the complex structure of xylan, various backbone degrading enzymes, including xylanase and *β*-xylosidase, and side chain cleaving enzymes, such as acetyl xylan esterases, arabinofuranosidases and feruloyl esterases are required to efficiently and completely break down the xylan. Among them, xylanase and *β*-xylosidase are the key enzymes for hemicellulose hydrolysis (Biely et al., 2016). Xylanase randomly degrade the internal *β*-1,4 backbone by converting xylan into oligosaccharides, while *β*-xylosidase further cleave oligosaccharides into xylose, and simultaneously prevent xylanases from being inhibited by the end products of their hydrolysis (Moreira and Filho, 2016). However, current works mainly focus on the synergy of xylanase and *β*-xylosidase at mild temperature. Our knowledge about high temperature xylan hydrolysis is limited.

Thermophilic microorganisms are a potential source of enzymes that can function at high temperature (Huang et al., 2015). To obtain the protein of interest in a rapid, easy and specific manner, heterologous expression in a well described expression system would be necessary. The Gram-positive *Bacillus subtilis* is an attractive host for heterologous production of recombinant proteins. Compared with *Escherichia coli* expression system, which cannot secrete the recombinant proteins out of the cell and occasionally accumulate the target proteins as inclusion bodies, *B. subtilis* is non-pathogenic and can release the target proteins directly into culture medium, greatly facilitating the downstream isolation and purification. *Bacillu subtilis*’ large-scale fermentation with high cell density and non-biased codon usage are other attractive features of the expression system (Zhao et al., 2020; Garg et al., 2023).

*Pseudothermotoga thermarum* isolated from continental solfataric springs in Lac Abbe, Djibouti is a hyperthermostable bacterium that can grow at 80°C, pH 5.5-9.0. The bacterium possesses a series of thermostable cellulosic degrading enzymes (Dipasquale et al., 2018). Previously characterized enzymes from *Pseudothermotoga thermarum* exhibited excellent thermostability at high temperature (>80°C) (Wagschal et al., 2016; Almeida et al., 2022). In the present work, thermostable xylanase and *β*-xylosidase genes from *Pseudothermotoga thermarum* were cloned and secretory expressed in *B. subtilis*, respectively. Biochemical properties and molecular docking of these xylanase and *β*-xylosidase were systematically evaluated. Then the synergistic action of these two enzymes on the hydrolysis of xylan and alkali-catalyzed glycerol pretreated lignocellulose at high temperature was studied. Our results showed that compared with *E. coli*, *B. subtilis* was a suitable candidate for secretory expressing the two enzymes, resulting in a 1.5 time increase in enzymatic activity. The synergistic activity of xylanase and *β*-xylosidase suggested possible application in biofuel and other industrial fields.

## 2 Materials and methods

### 2.1 Genes, growth media, and chemicals

The codon optimized xylanase gene Xyn and *β*-xylosidase gene XynB from *Pseudothermotoga thermarum* DSM5069 were synthesized by Genray Biotech Co., Ltd. (Shanghai, China). *Escherichia coli* DH5α and *Bacillus subtilis* WB800 were used for cloning and secretory expression of enzymes. *Escherichia coli* and *B. subtilis* were grown in LB media at 37°C. 100 μg/mL ampicillin for *E. coli* and 50 μg/mL kanamycin for *B.* subtilis were used for selecting transformed bacteria. Beechwood xylan and *p*-nitrophenyl-β-D-xylopyranoside (*p*NPX) were purchased from Sigma to analyze Xyn activity and XynB activity, respectively.

### 2.2 Construction of plasmid for secretory expression in *B. subtilis*

To construct a *E. coli/B. subtilis* shuttle vector on the basis of pWB980, DNA fragments of pWB980 and pET-20b were amplified by using primers shown in Table 1 (P1-P4). The amplified DNA fragments were digested with enzymes of *Nde*I and ligated together to obtain pWB980-20b. To secretory express Xyn and XynB in *B. subtilis*, the mature *Xyn* and *XynB* genes were amplified using P5-P6 and P7-P8 primers, respectively. The PCR products were digested with *Kpn*I and *Xba*I, and then ligated into pWB980-20b, generating pWB980-20b-*Xyn* and pWB980-20b-*XynB*. The cloning operations were conducted in *E. coli* DH5α.

**TABLE 1:** Primers used for construction of the plasmids.

Plasmid	Primer	Sequence (5^’^-3^’^)	Restriction site
pWB980	P1: pWB980-1	GGG​AAT​TCCAT​ATGCCC​CCC​TTT​GCT​GAG​GTG​GC	*Nde*I
	P2: pWB980-2	GGG​AAT​TCC​ATA​TGA​AAA​ATC​AGC​AAG​GGA​CAG​GTA	*Nde*I
pET-20b	P3: pET-20b-1	GGG​AAT​TCC​ATA​TGA​CAG​AAT​CAG​GGG​ATA​ACG​CAG	*Nde*I
	P4: pET-20b-2	GGG​AAT​TCC​ATA​TGC​GTT​TAC​AAT​TTC​AGG​TGG​CAC	*Nde*I
pWB980-20b-*Xyn*	P5: Xyn-1	CGG​GGT​ACC​ATG​GCA​GTT​GTG​GCA​AAC​TAC​GAT	*Kpn*I
	P6: Xyn-2	CTA​GTC​TAG​ACT​TAG​TCA​GGA​TCA​GGT​TGC​CCA​C	*Xba*I
pWB980-20b-*XynB*	P7: XynB-1	CGG​GGT​ACC​ATG​GAC​CTG​TAC​AAG​AAC​CCG​AAC	*Kpn*I
	P8: XynB-2	CTA​GTC​TAG​ATT​CGA​TTT​TGG​TGT​TAG​TAA​AAA​AC	*Xba*I

### 2.3 Expression and purification of Xyn and XynB in *B. subtilis*

The recombinant plasmids pWB980-20b-*Xyn* and pWB980-20b-*XynB* were transformed into the competent cells of *B. subtilis* WB800. The recombinant bacteria were grown in LB media containing 50 μg/mL Kanamycin at 37°C. The cells were harvested by centrifugation at 10,000 g for 10 min and then sonicated to release the intracellular proteins. The intracellular proteins and extracellular proteins were then heat treated at 60°C for 30 min and centrifuged (10,000 g for 20 min). The resulting supernatants were purified by affinity chromatography on Ä KTA*TPLC*™ with a HiTrap column (GE Healthcare Life Sciences). To confirm the target proteins’ purity, SDS-PAGE was used. Bradford method was used to measure the concentration of the purified enzymes.

### 2.4 Xyn and XynB assays

Under the standard assay condition, the recombinant enzyme was mixed with substrate beechwood xylan (20 mg) or *p*NPX (20 mM) in a final volume of 50 mM sodium acetate buffer (pH 5.0). The total reaction volume was 0.5 mL. For Xyn assay, 0.3 mL of 3,5-dinitrosalicylic acid (DNS) was added after incubation at 85°C for 15 min. The solution was boiled for 5 min and then measured at 550 nm. The XynB activity was stopped by adding 0.6 mL of 1M Na_2_CO_3_ and measured the absorbance at 405 nm. One unit of Xyn or XynB activity was defined as the amount of enzyme releasing 1 µmol reducing sugar or *p*NPX per minute. The values were given as the averages of three separate determinations.

To determine the effect of pH on enzyme activity, Xyn and XynB were incubated at different pH values (4.0-8.5) using 50 mM citrate buffer (pH 4-6.5) and 50 mM Tris-HCl buffer (pH 6-8.5), respectively. The pH stability of the two enzymes was determined by measuring the remaining activity after incubating the enzymes for 1 h at different pH values. The optimal temperature of Xyn and XynB was examined by standard assay ranging from 55°C to 100°C. The effect of temperature on the stability of the two enzymes was assessed by measuring the residual activity after pre-incubating enzyme at 75–95°C for 0.5 h. All measurements were performed in triplicate.

The influence of metal ions and chemicals on the enzyme activity was conducted by incubating the enzyme with 5 mM (final concentration) MgCl_2_, MnCl_2_, CaCl_2_, CuCl_2_, Tris, Tween 60, or EDTA for 1 h before adding substrate to start the reaction. All experiments were performed in triplicate.

### 2.5 Molecular docking

The 3D structure of Xyn and XynB was predicted using SWISS-MODEL, respectively. Each enzyme model was validated by different parameters, including Ramachandran’s plot and QMEAN scoring. PROCHECK was then used to further evaluate the results. The 3D structure of xylan substrate was obtained from PubChem. AutoDock was then used for docking study. According to amino acids involved in hydrogen bond formation and binding energy, appropriate docking results were selected. The docking results were finally visualized using PyMol (Thatoi et al., 2022).

### 2.6 Synergism between Xyn and XynB

To evaluate the synergism between Xyn and XynB, the hydrolysis experiments were investigated by combining Xyn and XynB in 1:1, 2:1, 4:1, and 8:1 ratios with 1% (w/v) beechwood xylan prepared in 50 mM citrate buffer (pH 6.0) at 80°C, 180 rpm in a bench top hybridization incubator. Three sets of hydrolysis were conducted, including Xyn/XynB alone or in combination hydrolysis. The amount of reducing sugars were measured using 3.5-dinitrosalicylic acid (DNS). The following equation was used to calculate the degree of synergism between Xyn and XynB:

$$\text{DS}=\frac{A_{\text{mixture}}}{{\sum A}_{\text{individual}}}$$

where *A*_mixture_ is the reducing sugar content achieved with Xyn and XynB added together, and *Σ*_individual_ is the sum of reducing sugar content released with the individual enzymes.

### 2.7 Enzymatic hydrolysis of pretreated lignocellulose

The hydrolysis experiment was carried out by hydrolyzing pretreated corncob and sugarcane bagasse at 10% (w/w) solids loading. The alkali-catalyzed glycerol pretreatment was conducted according to previously described procedures (Long et al., 2020a). The reaction mixtures were shaken at 180 rpm. Two-stage hydrolysis was conducted by first prehydrolyzing the substrates using thermostable Xyn and XynB at 85°C for 6 h, which was then followed with common hydrolysis by using Celluclast 1.5 L and Novozymes 188 at 50°C. Samples for sugar analysis were collected after 4, 8, 12, 24, 48 and 72 h of hydrolysis. Glucose concentration after hydrolysis was measured using HPLC equipped with an Aminex HPX-87H column. At the end of hydrolysis, samples were boiled at 100°C for 10 min to inactivate the enzymes. The supernatants were collected by centrifugation at 10,000 *g* for 10 min. All experiments were performed twice.

## 3 Results and discussion

### 3.1 Construction of a *B. subtilis*-*Escherichia coli* shuttle vector pWB980-20b

**FIGURE 1:**
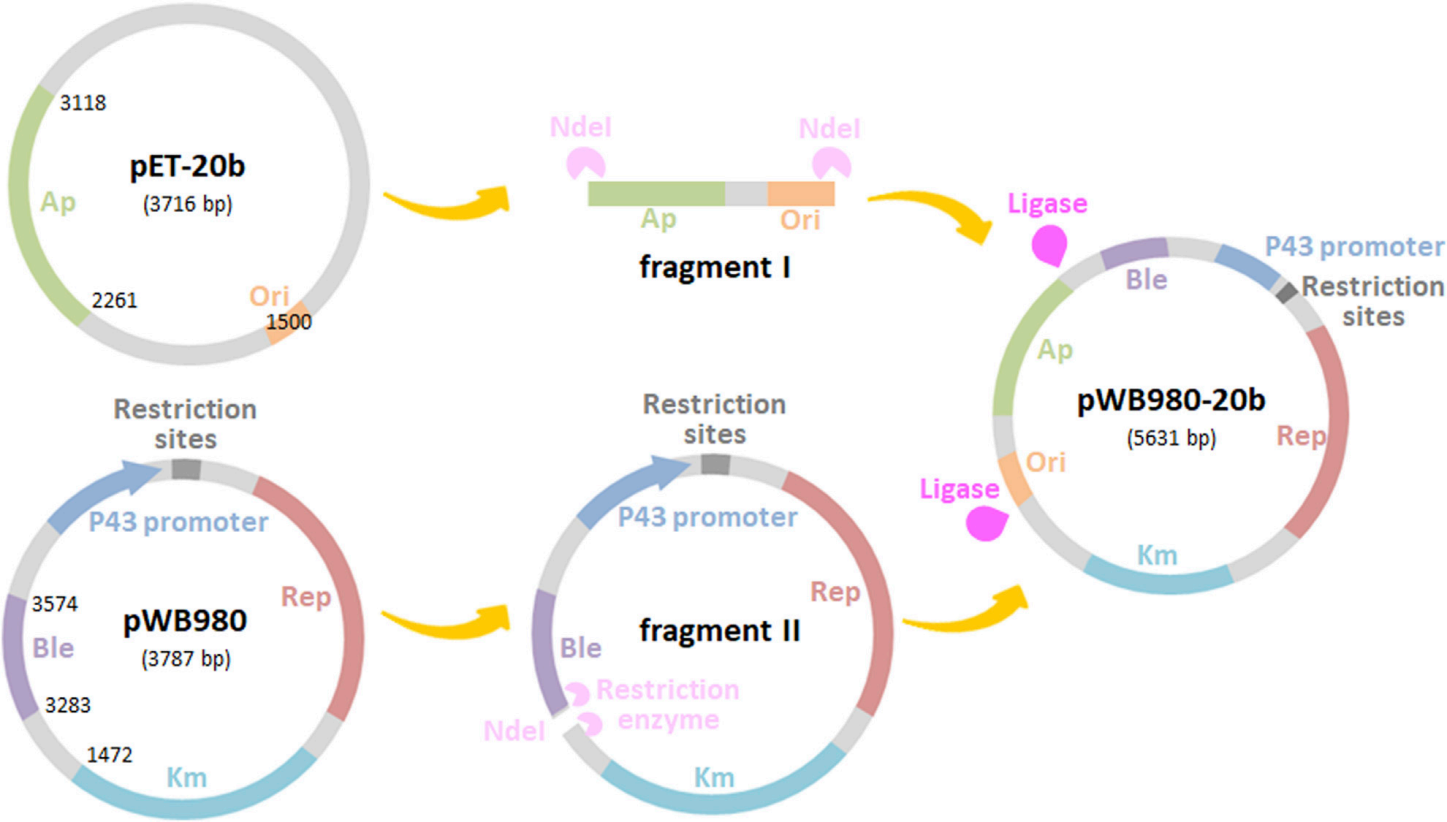
Construction scheme for shuttle vectors derived from pWB980. The pWB980-20b shuttle vector could replicate in both *E. coli* and *B. subtilis*.

*Bacillu subtilis* have been developed as one of the promising candidates for heterologous proteins secretion. The expression vector pWB980, derived from pUB110 is commonly used for producing foreign proteins due to its high copy number and high stability. However, the transformation efficiency of *B. subtilis* as the primary host is low, limiting the application of pWB980 (Zhao et al., 2020). To improve the transformation efficiency of this plasmid, a new *B. subtilis-E. coli* shuttle vector pWB980-20b was first constructed on the basis of pWB980 in this study. The process of construction was shown in Figure 1. A 1841 bp fragment containing Ap^R^ gene and the *ori* for *E. coli* was amplified from the Gram-negative plasmid pET-20b by PCR, resulting in fragment I. The P43 promoter, Km^R^ gene, *ble,* and the plus *ori* for *B. subtilis* were amplified from pWB980 by reverse transcriptase PCR, resulting in fragment II. After digestion with *Nde*I and ligation of the two resulting fragments, shuttle vector pWB980-20b was constructed. pWB980-20b was in the size of 5,631 bp. With introduction of the *ori* for *E. coli*, pWB980-20b was capable of conducting the initial cloning steps in *E. coli* and then transferring the recombinant plasmid to *B. subtilis*. Transformants with this plasmid can be either selected with Ampicillin in *E. coli* or Kanamycin in *B. subtilis*.

### 3.2 Cloning, expression and purification of recombinant Xyn and XynB

**FIGURE 2:**
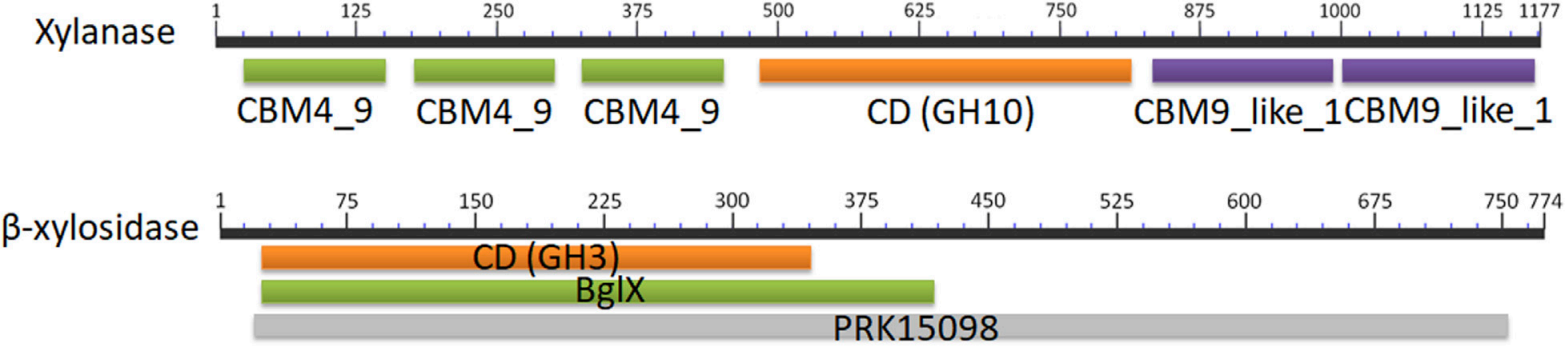
Primary structure of the xylanase and the *β*-xylosidase from *P. thermarum*. The structure of each enzyme was identified by BLAST. CBM4_9, family 4_9 carbohydrate binding module; GH10, family 10 catalytic domain; CBM9_like_1, family 9_like carbohydrate binding module; GH3, family 3 catalytic domain; BglX, *β*-glucosidase-related glycosidase; PRK15098, *β*-D-glucoside glucohydrolase.

To express the xylanase (Xyn, Genbank No. WP_013932897) and the *β*-xylosidase (XynB, Genbank No. WP_013931466) from *Pseudothermotoga thermarum* DSM5069 in *B. subtilis*, the DNA fragments of *Xyn* and XynB genes, encoding 1,177 and 774 amino acids with predicted p*Is* of 5.31 and 6.12, were amplified from the optimized synthetic fragments, respectively. Xyn was a modular protein containing three N-terminal carbohydrate binding domains (CBMs), two C-terminal CBMs, and a catalytic domain (CD) (Figure 2). The CD (L487-K812) displayed considerable similarity to catalytic domains of glyosidic hydrolase family 10 (GH10). XynA from *Thermotoga* sp. Mc24 (Genbank No. WP_038052050) exhibited the highest homology (66.4%). The N-terminal noncatalytic region of Xyn contained three tandem family 4_9 CBMs. Teo et al. reported that the presence of CBM4_9 in xylanase from *Roseithermus sacchariphilus* resulted in an increase in NaCl tolerance (∼17%) and turnover rate (∼40%) of the catalytic domain (Teo et al., 2019). Kim et al. found that the CBM4_9 family module in 1,4-β-xylanase KRICT PX3 from *Paenibacillus terrae* HPL-003 was essential for assisting the hydrolysis of insoluble xylan by promoting the binding to xylan (Lim et al., 2016). The C-terminal domain contained two tandem CBM9s, which were located at G828-E987 and G992-T1176. CBM9 played a role in driving the enzymes to their targeted insoluble cellulose and soluble sugars, including soluble hexose sugars, xylan and glucopyranoside-based polymers (Boraston et al., 2001). The *β*-xylosidase from *P. thermarum* in this study included a specific glycosyl hydrolase family 3 (GH3) domain. Its non-specific hits included a N-terminal domain *β*-glucosidase-related glycosidase (BglX). These elements might form the *β*-D-glucoside glucohydrolase (PRK15098).

**FIGURE 3:**
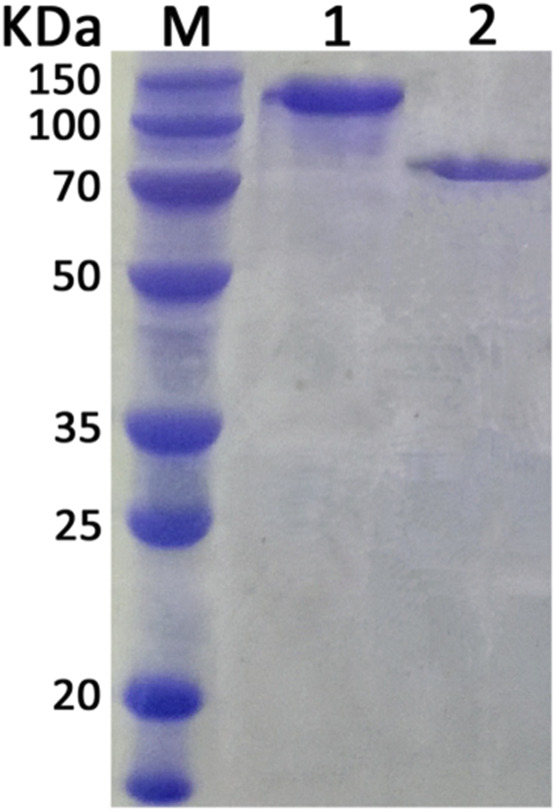
SDS-PAGE analysis of purified enzymes. Lane M, protein marker. Lane 1, purified xylanase (Xyn). Lane 2, purified *β*-xylosidase (XynB).

Expression plasmids pWB980-20b-*Xyn* and pWB980-20b-*XynB* were then constructed and transformed into *B. subtilis* WB800 to express the enzymes, respectively. To measure the expression level of Xyn and XynB, enzyme activities of the intracellular and extracellular fractions of the recombinant bacteria were determined (Table 2). As shown in Table 2, the total enzymatic activity was 147.2 U/mL for Xyn and 220.8 U/mL for XynB. Both of the enzymatic activities of the two enzymes expressed in *B. subtilis* were approximately 1.5 times as high as those expressed in *E. coli* (94.3 U/mL for Xyn and 150.3 for XynB), indicating that *B. subtilis* was a potential candidate to achieve a high level expression of the enzymes for subsequent application. The recombinant proteins were then purified using Ni^2+^ affinity chromatography after being heated at 60°C for 30 min. The purified Xyn and XynB showed single bands on SDS-PAGE with molecular masses of about 130 kDa and 85 kD, respectively, which were identical to their calculated masses, stating that the target proteins were successfully expressed in *B. subtilis* (Figure 3).

**TABLE 2:** Expression of the recombinant Xyn and XynB in *B. subtilis* WB800.

Enzyme	Intracellular activity (U/mL)	Extracellular activity (U/mL)	Total activity (U/mL)
Xyn	24.1 ± 0.6	123.1 ± 1.6	147.2 ± 1.1	
XynB	35.7 ± 1.3	185.1 ± 3.9	220.8 ± 5.2	

### 3.3 Biochemical properties of Xyn and XynB

**FIGURE 4:**
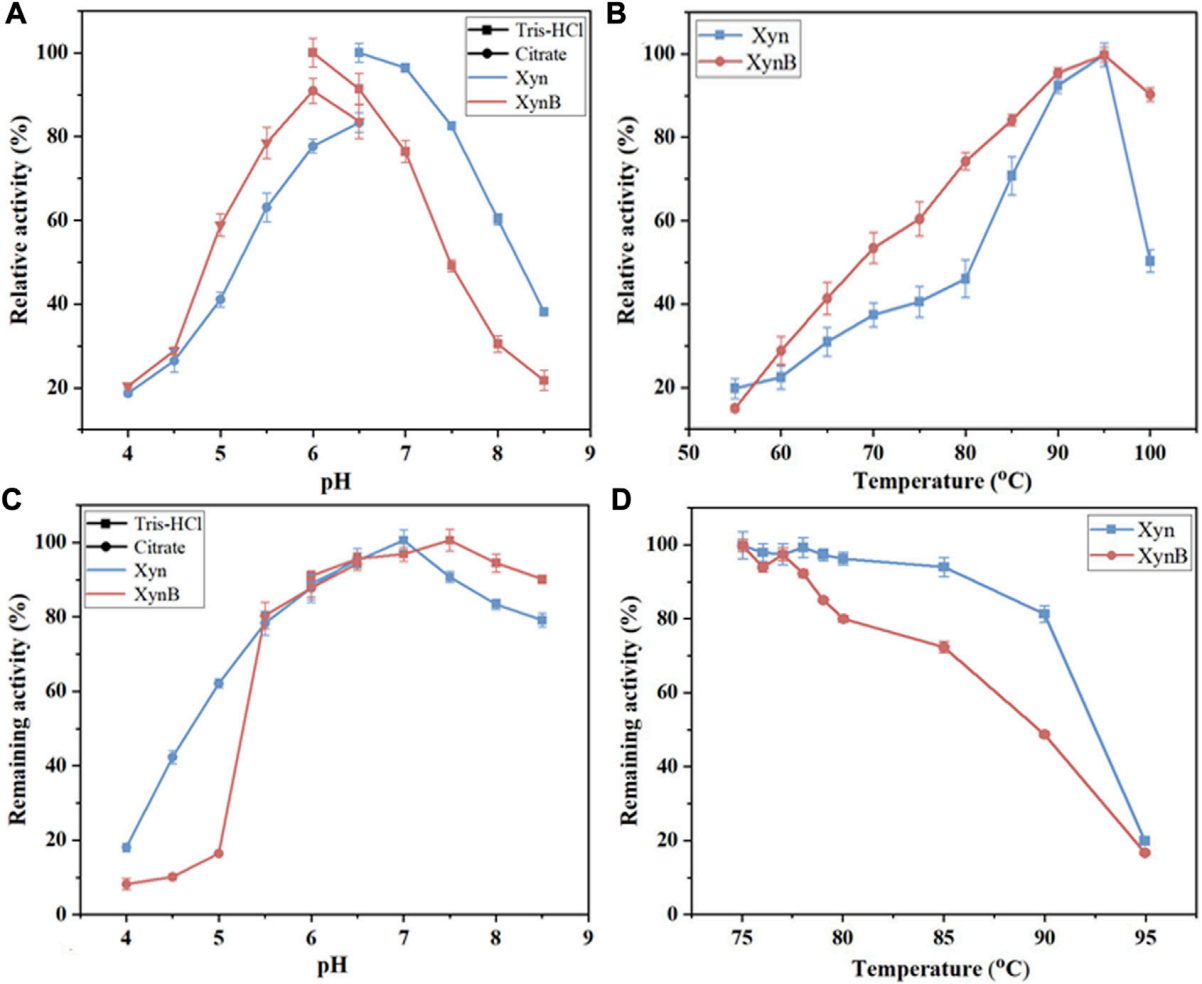
Effects of temperature and pH on the activity and stability of the recombinant Xyn and XynB expressed in *B. subtilis* WB800. **(A)** Optimal pH. **(B)** Optimal temperature. Citrate (pH 4.0-6.5) and Tris-HCl (pH 6.5-8.5) are reaction buffers. **(C)** pH stability. **(D)**, Remaining activity of Xyn with 5 mM Ca^2+^ and XynB at different temperatures ranging from 75°C to 95°C. Values shown were the means of triplicate experiments.

After purification, biochemical properties of the enzymes (*e.g.*, optimal pH, optimal temperature, and pH thermostability) were tested systematically to determine the optimal conditions for the subsequent synergistic hydrolysis. It showed that the purified Xyn exhibited maximal activity at pH 6.5 and 95°C (Figures 4A, B). The Xyn was stable between pH 5.5 and 8.5, retaining >80% of the initial activity after 2 h of incubation at 85°C. When pH < 5.5, the enzyme was rapidly inactivated (Figure 4C). As for the temperature thermostability, Xyn retained more than 80% of its activity after incubating with 5 mM Ca^2+^ at 90°C for 0.5 h, however, it was observed that this enzyme was unstable at its optimal temperature (95°C) and lost ∼80% of its activity after incubation for 0.5 h (Figure 4D). When various additives were added into the reaction respectively, the activity of Xyn was not affected by MgCl_2_, AlCl_3_, Tween 60 and EDTA, but slightly affected by Tris. The activity of Xyn was significantly boosted by CaCl_2_ (Table 3). With 5 mM of CaCl_2_ addition, the Xyn activity was greatly enhanced by 394%. However, Xyn activity was strongly inhibited by CuCl_2_, only remaining ∼10% of its activity in the solution. This was due to Cu^2+^ could not only bind thiol groups of amino acids, but also interact with their carboxyl groups or imidazole (Yin et al., 2010). These results suggested that Ca^2+^ might play a role in keeping the enzyme in a catalytically active conformation.

**TABLE 3:** Effects of reagents on Xyn and XynB activities.

Reagents^a^	Relative activity (%)	
	Xyn	XynB
Control^b^	100 ± 0.4	100 ± 0.5
MgCl_2_	112 ± 2.5	102 ± 3.7
MnCl_2_	227 ± 2.4	105 ± 1.6
CaCl_2_	394 ± 2.7	104 ± 3.1
CuCl_2_	10 ± 0.5	4.6 ± 0.2
ZnCl_2_	40 ± 0.7	69 ± 1.7
CoCl_2_	59 ± 0.8	87 ± 0.9
AlCl_3_	92 ± 1.4	87 ± 1.2
Tris	81 ± 1.9	102 ± 2.0
Tween 60	107 ± 3.2	97 ± 3.4
EDTA	102 ± 0.9	101 ± 0.8

^a^ The final concentration of metal ions was 5 mM. the final concentrations of tris, Tween 60, and EDTA, were 0.05%, 0.05%, and 0.5 mM, respectively. Experiments were performed in triplicate.

^b^ Enzyme activity without adding metal ions and chemical reagents was defined as 100%.

The optimal pH and temperature for purified XynB were 6.5 and 95°C (Figures 4A, B), which possessed the general properties of previously reported *β*-xylosidase that were more active in slightly acidic solution (pH 4.5-6.5) (Wang and Arioka, 2021; Mercado-Flores et al., 2022; Zafar et al., 2022). The purified XynB was stable over a wide range of pH (5.5-8.5), retaining >80% of its initial activity after 2 h of incubation at 80°C (Figure 4C). When the temperature increased, the remaining activity of XynB decreased rapidly. Similar to Xyn, XynB was also unstable at its optimal temperature (95°C), only retaining ∼20% of the initial activity. However, at 80°C, it showed approximately 80% of its initial activity (Figure 4D). The effects of additives on XynB activity were also investigated (Table 3). XynB activity was strongly inhibited by CuCl_2_, but not affected by most of the additives, including MgCl_2_, MnCl_2_, CaCl_2_, Tris, Tween 60 and EDTA. ZnCl_2_, CoCl_2_ and AlCl_3_ slightly inhibited XynB activity. The capacity to resist these metal ions and chemical reagents suggested that XynB could survive in a variety of scenarios.

### 3.4 Docking studies of modeled Xyn and XynB with substrate

To further understand the properties of the two enzymes, molecular docking analysis of modeled enzymes with substrates was also conducted. The predicted 3D models of Xyn and XynB were obtained by the SWISS-MODEL, respectively. Both predicted Xyn and XynB models showed >95.0% residues in Ramachandran’s favorable region with QMEAN score less than 2 (Supplementary Figure S1). These results indicated that the predicted models were of good quality and could be used for docking.

**FIGURE 5:**
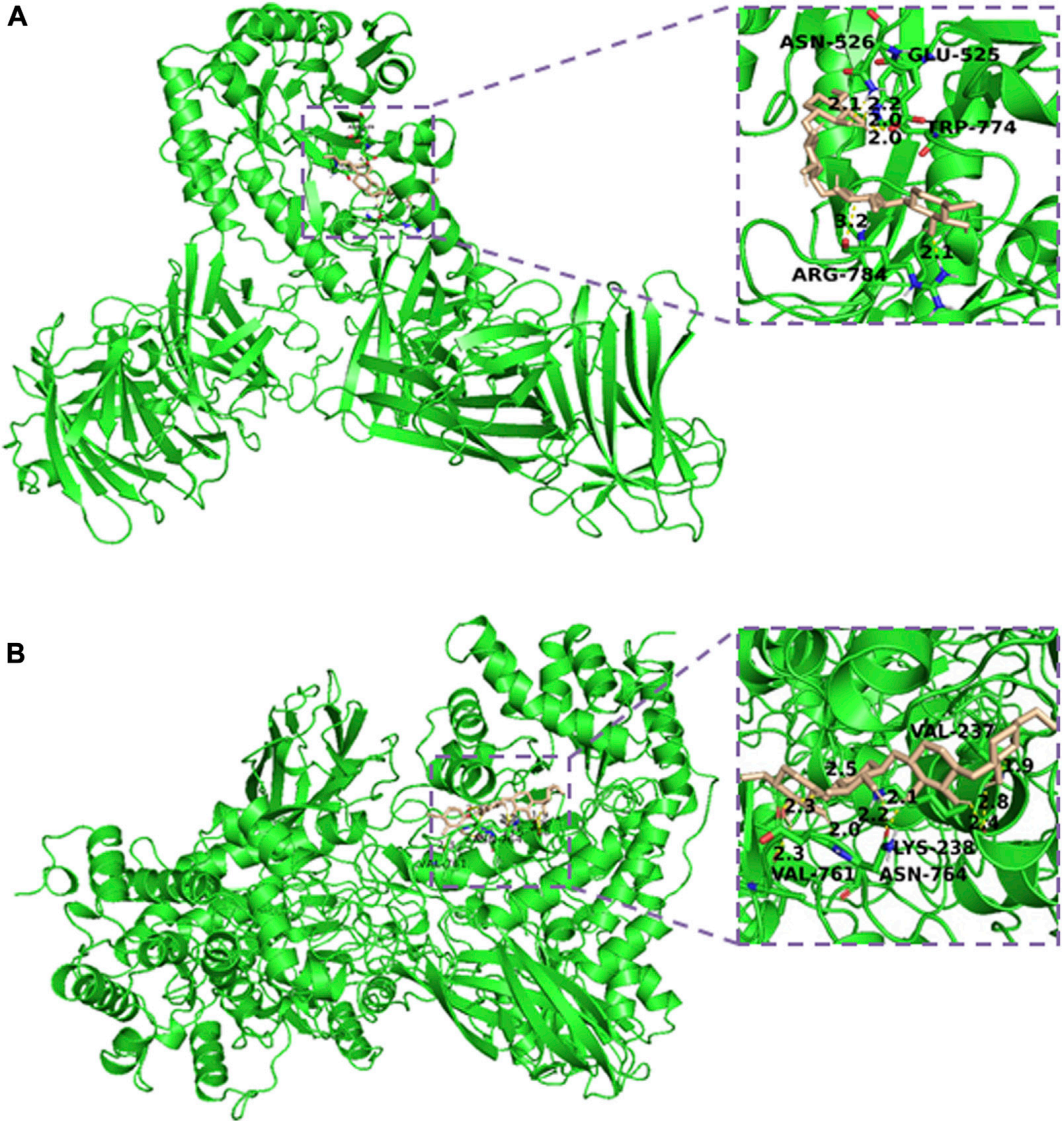
Interaction model of xylan and Xyn **(A)** /XynB **(B)** produced by Autodock 4. The 3D structure of each enzyme was constructed by SWISS-MODEL.

The predicted 3D models Xyn and XynB were then docked with the substrate xylan by AutoDock software. The complex with binding affinity less than −5 kcal/mol was selected for the interaction studies. Figure 5A showed the interaction between Xyn and the substrate xylan. It appeared that Glu525, Asn526, Trp774 and Arg784 were all involved in the binding and catalysis of xylan. The bond length between residues and substrate was 2.2, 2.1, 2.0 and 2.1, respectively. These docking results suggested that Glu525, Asn526, Trp774 and Arg784 were critical for Xyn-xylan interaction. The complex was further stabilized by the surrounding hydrophobic amino acids, resulting in the further improvement of enzyme activity and thermostability (Saleem et al., 2021). Compared with the molecular docking results of the xylanase from *Thermotoga maritima*, same residues were involved in the binding interaction between enzyme and xylan substrate (Yang and Han, 2018). However, the docking results of xylan with xylanase from *Geobacillus thermodenitrificans* C5 showed the involvement of Glu, Pro, Arg, Trp and His (Irfan et al., 2018). The interaction of xylopentose with xylanase from *Thermotoga petrophila* RKU1 analyzed through molecular docking also involved Glu525, Asn526 and Trp774 residues with substrate (Shahid et al., 2023). However, residues position is different for all these xylanases.

Additionally, molecular docking study of the XynB-xylan complex was carried out using AutoDock, and the results were shown in Figure 5B. The fissure structure of XynB provided favorable conditions for the protein to release the binding of small molecule substrate. It showed that Val237, Lys238, Val761 and Asn764 were all involved in the binding and catalysis of xylan. Lots of hydrogen bonds were formed by these residues, endowing the enzyme with favorable heat resistance during hydrolysis. Generally, stable hydrogen bonds were formed at distances less than 3.5 Å. The distances of residues to xylan were observably shorter at 1.9-2.8 Å, suggesting that a stably bound XynB-xylan complex. It was reported that heat resistant ability of the enzyme was correlated with the number of hydrogen bonds formed in the reaction (Liu et al., 2023). However, compared with Xyn, which had higher heat resistant ability with fewer hydrogen bonds than that of XynB, indicating that there were still other factors might also influence enzyme thermostability (Nezhad et al., 2022). These results showed some key residues’ information of both enzymes, which could further instruct enzyme directed evolution to improve their properites.

### 3.5 Synergistic effect of Xyn and XynB on xylan degradation

**FIGURE 6:**
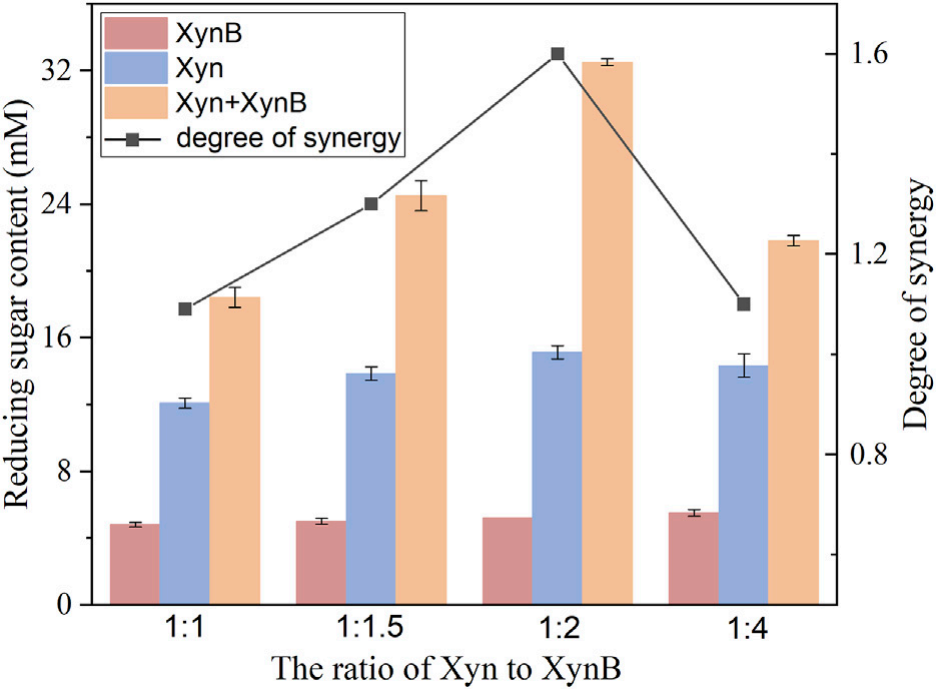
Synergistic hydrolysis of beechwood xylan by Xyn and XynB.

To investigate the synergism of Xyn and XynB, varying ratios were incubated with beechwood xylan. The resulting saccharides were determined by DNS. As shown in Figure 6, Xyn exhibited higher activity than XynB when hydrolyzing beechwood xylan. The reducing sugar concentrations were 18.4 mM for Xyn and 4.8 mM for XynB.

When combining the Xyn and XynB together for xylan hydrolysis, a significant synergistic effect was observed with a considerably increased reducing sugar content. When different Xyn: XynB ratios were used, the degree of synergy was 1.1 (1:1), 1.3 (1.5:1), 1.6 (2:1) and 1.1 (4:1), respectively. Combining the two enzymes in a 2:1 ratio liberated the most reducing sugar from xylan. The total amount of reducing sugar released was about 53% higher than that of Xyn alone. These results were similar to those reported previously, which also observed the synergistic effect of xylanase and *β*-xylosidase on xylan deconstruction (Zhou et al., 2012; Yang et al., 2014; Fanchini Terrasan et al., 2016). Similar to cellulose degradation, hydrolysis of xylan also required the cooperation of enzymes to work synergistically. Xylanase initially generated soluble oligosaccharides from xylan by directly cleaving glycosidic bonds. The released oligosaccharides were further converted to xylose by *β*-xylosidase, and simultaneously reduced the inhibition of xylanase by its reaction products (Van Dyk and Pletschke, 2012). Since shorter oligosaccharides are more favorable for generating xylose by *β*-xylosidase, xylanase requirement is higher than that of *β*-xylosidase to achieve a high hydrolysis yield. Notably, the addition of Xyn presented a nonmonotonic effect on reducing sugar content for xylan hydrolysis, which was likely due to the acceleration of Maillard reaction by increasing protein loading (Wei et al., 2018). Thus, an over-high addition of enzymes was unfavorable to high temperature hydrolysis.

### 3.6 Synergistic effect of Xyn and XynB on lignocellulose degradation

**FIGURE 7:**
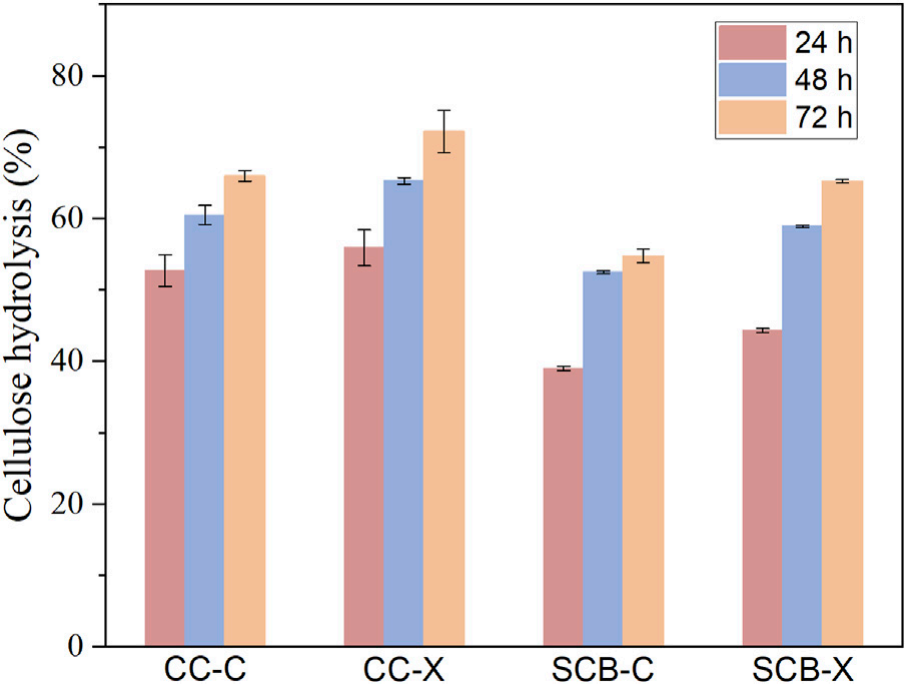
The extent of cellulose hydrolysis of pretreated corncob (CC) and pretreated sugarcane bagasse (SCB) at 10% solid loading after 24, 48 and 72 h. C, substrate was incubated at 80°C for 6 h without Xyn and XynB addition. X, hydrolysis in the presence of Xyn and XynB with a ratio of 2:1 of 1^st^ stage at 85°C for 6 h.

Since the recombinant Xyn and XynB exhibited obvious synergistic hydrolysis of model substrate (beechwood xylan), plus the commercial cellulase was active only at mild temperature, we next evaluated the synergistic effect of these two enzymes on hydrolysis of pretreated corncob (CC) and sugarcane bagasse (SCB) using two-stage hydrolysis. Chemical compositions of CC and SCB after pretreatment were shown in Table 1. It was apparent that Xyn and XynB pre-hydrolysis could facilitate the hydrolysis yield on both CC and SCB substrates, but the extent of improvement was highly substrate dependent (Figure 7). For example, although CC and SCB substrates contained same amount of xylan, the thermostable Xyn and XynB treatment could improve cellulose conversion by 11% for the CC substrate, while a less improvement (7%) was obtained on the SCB substrate. This was likely caused by the highly branched xylan structure of the SCB restricted the accessibility to enzymes. In addition, it appeared that the hydrolysis boosting effect of Xyn and XynB treatment was much higher at the first 24 h, which was probably due to the increased accessibility of cellulose after Xyn and XynB treatment.

## 4 Conclusion

In this study, the thermostable Xyn and XynB from *Pseudothermotoga thermarum* were effectively secretory expressed in *B. subtilis* WB800. The recombinant enzymes were successfully purified and determined by SDS-PAGE. Compared with the enzymes expressed in *E. coli*, the two enzymes expressed in *B. subtilis* resulted in a 1.5 time increase in enzymatic activities. The Xyn and XynB possessed higher thermostability compared with the characteristics of enzymes from other microorganisms. The molecular docking analysis of the two enzymes, via AutoDock software, suggested amino acid residues that were involved in enzyme-ligand interaction. In addition, Xyn and XynB exhibited synergistic hydrolysis of beechwood xylan, where the degree of synergy was 1.6. This study indicated the promising potential of using thermostable Xyn and XynB for hemicellulose deconstruction, making them suitable candidates for the existing enzymatic hydrolysis. Moreover, this study also paved the way for tailoring more effective enzyme cocktails with additional thermostable enzymes to further facilitate lignocellulose deconstruction.

## Data Availability

The original contributions presented in the study are included in the article/Supplementary Material, further inquiries can be directed to the corresponding author.

## Abbreviations

Xyn, xylanase; XynB, xylosidase.
