# Supplementary material for: Improved secretory expression and characterization of thermostable xylanase and β-xylosidase from Pseudothermotoga thermarum and their application in synergistic degradation of lignocellulose
Source: Front Bioeng Biotechnol. 2023 Sep 18;11:1270805. doi: 10.3389/fbioe.2023.1270805 (PMC10544939; doi:10.3389/fbioe.2023.1270805)
Supplement: Supplementary file 1 [file DataSheet1.docx]

**Improved secretory expression and characterization of thermostable xylanase and β-xylosidase from *Pseudothermotoga thermarum* and their application in synergistic degradation of lignocellulose**

**Jinkang Chen^1^, Hao Qin^2,3^, Lingfeng Long^1^**^*^

^1^Key Laboratory of Industrial Biotechnology, Ministry of Education, Jiangnan University, Wuxi 214122, China

^2^Eco-materials and Renewable Energy Research Center (ERERC), College of Engineering and Applied Sciences, Nanjing University, No. 22, Hankou Road, Nanjing, Jiangsu 210093, China.

^3^Little Swan Electric Co. Ltd, Midea Group, Wuxi 21400, China

*** Correspondence:**Lingfeng Long
lingfengdragon@jiangnan.edu.cn


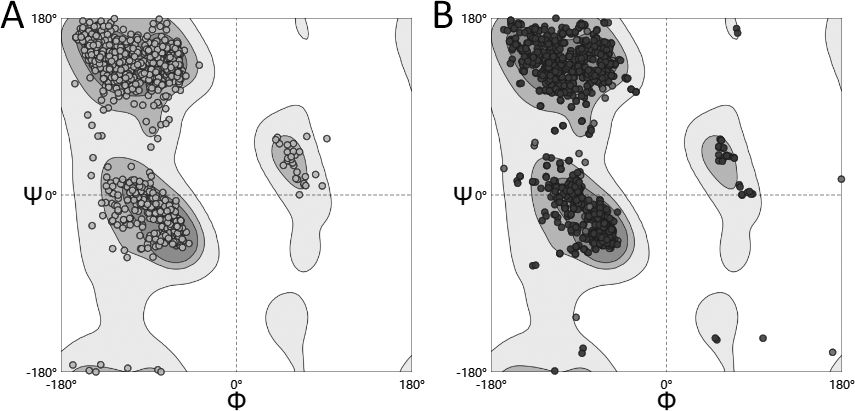


**Figure S1**. Analysis of Ramachandran’s plot by PROCHECK with 96.8% residues in the most favored regions for the predicted Xyn (A) and 95.8% residues for the predicted XynB (B).

**Table S1** Chemical composition of alkali-catalyzed glycerol pretreated corncob (CC) and sugarcane bagasse (SCB)

| Feedstock | Chemical composition (%) | | |  |
| --- | --- | --- | --- | --- |
|  | Cellulose | Hemicellulose | Lignin | |
| Corncob | 52.0 ± 0.6 | 30.5 ± 1.2 | 8.3 ± 0.7 | |
| Sugarcane bagasse | 55.0 ± 0.3 | 31.9 ± 0.4 | 10.3± 1.2 | |
